# Sex estimation by volumetric evaluation of the maxillary canine using cone-beam computed tomographic images

**DOI:** 10.1186/s12903-024-03962-y

**Published:** 2024-02-06

**Authors:** Ceren Özeren Keşkek, Hümeyra Özge Yılancı, Gökçen Akçiçek

**Affiliations:** 1 Department of Dentomaxillofacial Radiology, Faculty of Dentistry, Izmir Democracy University, İzmir, Turkey; 2 Department of Dentomaxillofacial Radiology, Faculty of Dentistry, Hacettepe University, Ankara, Turkey

**Keywords:** Sex determination, Teeth, Maxillary canine, Cone-beam computed tomography, Forensic sciences

## Abstract

**Background:**

Teeth can be a reasonable part of sex estimation in case of being the single evidence available or in doubt although they are not as accurate as bones in sex estimation. The size of teeth is greater in males than in females. In this study, it was aimed to determine cut-off values of the tooth volume (TV) and root volume (RV) of the maxillary canines and to evaluate the validity of the method for discriminating between males and females.

**Methods:**

Cone beam computed tomography (CBCT) images of 250 individuals aged 18 to 63 years with equal age and sex distribution were assessed retrospectively. The sample divided into reference set including 168 images of 84 females and 84 males and validation set including 82 images of 48 females and 34 males. Receiver operating characteristic (ROC) curve analysis, and Youden’s index were used to determine cut-off values of the volumetric measurements and to test the discriminative performance of the method.

**Results:**

The optimal cut-off values of TV ≥ 581 mm^3^ and RV ≥ 334 mm^3^ for estimating sex were established from the reference set. The sensitivity (Se), specificity (Sp), and accuracy (Ac) were 0.77 for TV ≥ 581 mm^3^ while they were 0.82, 0.77, and 0.79 for RV ≥ 334 mm^3^. When we tested the previously derived cut-off values of TV of the maxillary canine, the Se, Sp, and Ac were found to be respectively 0.71, 0.90, and 0.82 for TV ≥ 619 mm^3^ and 0.97, 0.27, and 0.56 for TV ≥ 510 / 460 mm^3^ (right/left).

**Conclusions:**

The identified cut-off values of TV and RV of the maxillary canines can be used to estimate sex with moderate accuracy when the only evidence available is teeth or in case of doubt. The method’s applicability should be assessed for different populations.

## Introduction

Dental morphological and morphometric features provide valuable biological and cultural insights into individuals or human populations. Sex estimation serves as the initial step in establishing the biological profile of skeletal remains in forensic sciences and anthropology. There are various morphological and biochemical methods for sex estimation. The biochemical analysis of teeth, based on DNA and Barr bodies, offers the highest accuracy for sex estimation. However, it has limitations such as high cost, time-consuming processes, equipment requirements, and an invasive procedure that involves the destruction of the sample. Additionally, these methods may encounter challenges in cases of insufficient preservation of remains or limited DNA extraction from the sample. In contrast, morphological methods based on sex differences in bones and teeth are non-invasive, comparatively easier, faster, and more cost-effective than biochemical methods, proving advantageous, particularly in instances of mass disasters. Teeth are resistant to environmental conditions as the hardest structures in the body. Although teeth give less accurate results in sex estimation than bones, they are valuable tools for use in case of burning, purification, and fracture [1–5].

Many studies have been carried out using tooth size as an indicator for sex estimation and various methods have been proposed. In these studies, morphometric features of the teeth were usually assessed with linear measurements and the canines and subsequently the molars were found to be the most sex-differentiated permanent teeth [3, 6]. The accuracy of odontometric techniques for sex estimation has been reported around 80% in general [1, 2]. In the last decade, studies have been carried out on whether better results in sex estimation can be obtained by volumetric measurements using various tomographic imaging techniques and software programs, and with different parameters and statistical approaches [1, 7–13]. Tardivo et al. [1], using computed tomography (CT) images, found the accuracy of logistic regression models constructed with tooth volume of the canines between 82 and 86%. They reported the cut-off values of tooth volume for the right and left maxillary canines as 0.510 cm^3^ and 0.460 cm^3^, respectively. Kazzazi et al. [13] measured the root volume using CT and found the highest percentage of sexual dimorphism in the maxillary second incisor followed by the maxillary canine. They reported the accuracy of the discriminant functions between 80 and 100%. Manhaes-caldas et al. [7] measured the crown volume of the maxillary central, the maxillary and mandibular canines and the mandibular laterals using cone beam computed tomography (CBCT) and achieved an accuracy of 59–84%. In another study using CBCT, De Angelis et al. [12] proposed a simple method for sex estimation. They found the accuracy of sex discrimination based on the threshold value (0.619 cm^3^) of tooth volume of the maxillary canines to be 80.5%. In studies using micro-CT and discriminant analysis, an accuracy of 60–96% with various volumes and ratios of the maxillary and mandibular canines [8, 9], and between 66 and 90% with various volumes and surface areas of the maxillary and mandibular anterior and premolar teeth have been reported [10]. In another study on micro-CT, Sorenti et al. [11] found the accuracy of the logistic regression models derived by various area and surface ratios of the mandibular molars as 74.36%.

In this study, it was aimed to determine the diagnostic cut-off values of the volumetric measurements of the maxillary canines on CBCT images for sex estimation and to test the validity of the method.

## Methods

Our investigational protocol was approved by the Non-Interventional Clinical Research Ethics Board at İzmir Demokrasi University (Approval no. 2023/03–20). The study was performed on 250 CBCT images of 118 females and 132 males, aged 18 to 63 years, from the archive of the Department of Oral and Maxillofacial Radiology at Hacettepe University Faculty of Dentistry, recorded between January 2017 and December 2021. Study population was consisted of contemporary Turkish people from Middle Anatolia in Turkey. Turkey is situated in one of the most diverse regions of Europe and Asia due to the complex genetic background and widespread presence of Turkish people in both the Near East and Europe [14]. Hacettepe University Faculty of Dentistry is a state hospital that can receive patients from all regions of Turkey, with a particular emphasis on the central parts.

Images were selected, ensuring an equal sex and age distribution. Age and sex information of the individuals were recorded. Other inclusion criteria for the study encompass the following:

- The images should have diagnostic quality and be free from factors such as artifacts that could affect measuring.
- There should be at least one maxillary canine present on the right or left side.
- The canines should have completed apical development.
- The canines should be free of caries, notably attrition involving dentin, restoration, fracture, resorption, shape or size anomalies, or pathology.
- No maxillofacial anomalies or deformities should be present.

All images were randomly divided into two samples; reference set (N:168) and validation set (N:82). The reference set was constructed, ensuring the number equality of subjects per sex in three age groups, then, the validation set consisted of the remaining subjects. Table 1 shows age and sex distribution of the subjects for both samples.

**Table 1:** The number of the subjects according to the age groups and sex for both datasets

	*Reference set*			*Validation set*		
*Age Groups*	*Female*	*Male*	*Total*	*Female*	*Male*	*Total*
*18–29 years*	28	28	56	18	10	28
*30–44 years*	28	28	56	16	16	32
*45–63 years*	28	28	56	14	8	22
*Total*	84	84	168	48	34	82

All CBCT images were acquired with a CBCT device (i-CAT Next Generation, Imaging Sciences International, Hatfield, PA, USA) with a tube voltage of 120 kVp with variation in the field of view, acquisition voxel size, tube current (3–7 mA), and exposure time.

**Fig. 1:**
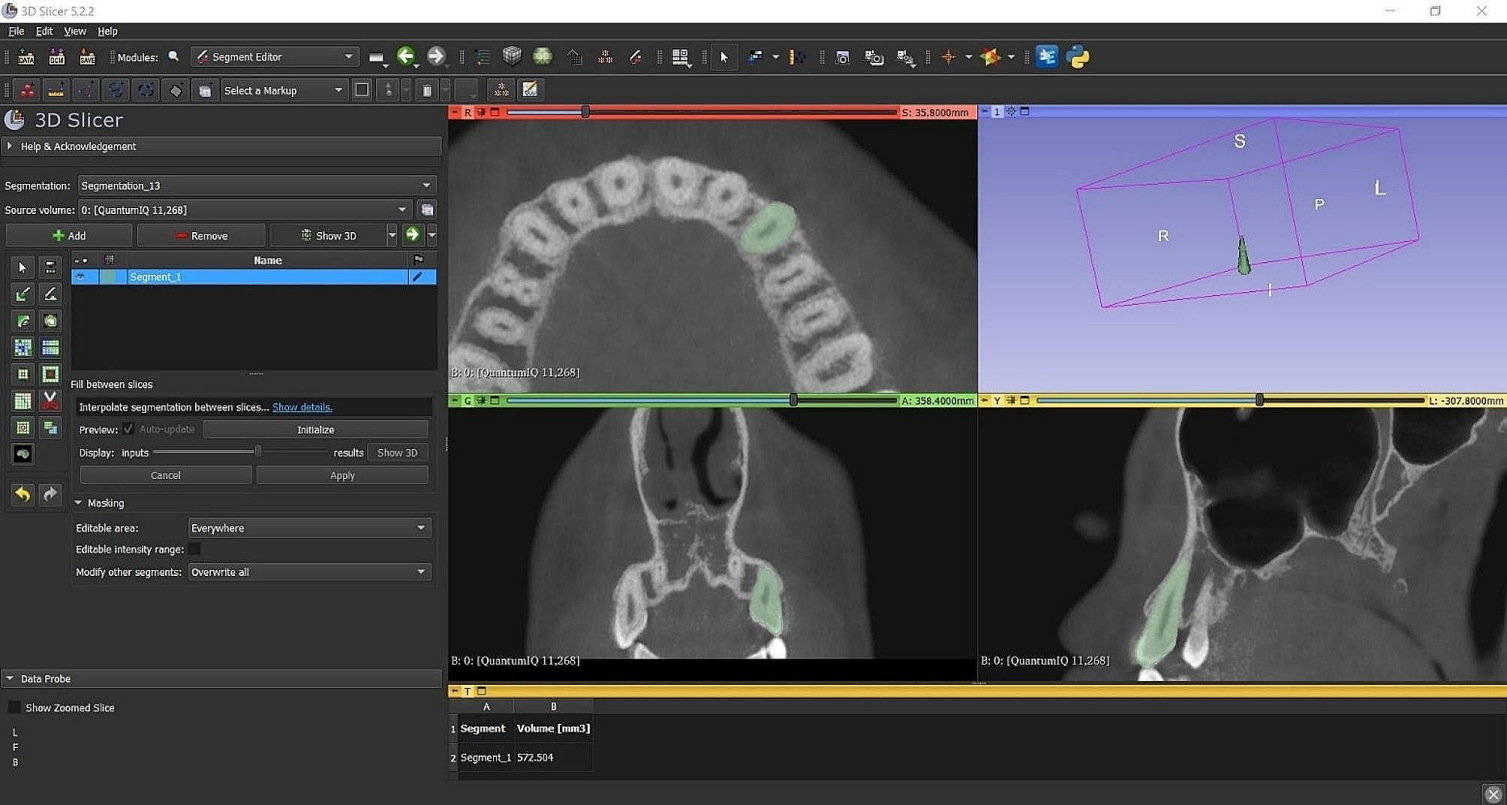
Volume measurement sample of the maxillary canine (3D Slicer Software, Segment Editor module)

The measurements were conducted on the left maxillary canines. If the left maxillary canine was not present, the right-side canines were used. The images were numbered to provide observer blinding during measuring. For the volumetric measurements of canines, CBCT images were transferred to 3D Slicer version 5.0.3 software (Surgical Planning Lab, Harvard Medical School, Harvard University, Boston, MA, USA). In the “Segment editor module”, each canine tooth was manually defined in the axial section and evaluated by creating a ROI (Region of interest) in every 2–3 slices from the crown to the root apex. Missing ROIs were created with the “filling between sections” feature in the program, and thus 3D models of the teeth were obtained. The “Segment statistics module” in the program was used to calculate the volumes in these models. The tooth volume (TV) and root volume (RV) of canine teeth were evaluated in mm^3^ (Fig. 1). All the measurements were carried out by a single observer - oral and maxillofacial radiologist. To assess intra-observer agreement, measurements were repeated on 24 randomly selected images two weeks after the initial measurements. To explore inter-observer agreement, the same 24 images were evaluated by another observer - oral and maxillofacial radiologist.

The statistical analysis of the data was performed using IBM SPSS (ver. 20) and easyROC web tool [15]. The significance level for the analysis was set at 5%. Intra-observer and inter-observer agreements were evaluated using the intra-class correlation coefficient (ICC).

To determine which statistical method to be used in determining the difference between the averages of the independent groups, the suitability of the data to the normal distribution was first tested with the Kolmogorov-Smirnov and Shapiro Wilk tests, and it was observed that the variables were not normally distributed. Spearman correlation coefficient was used for the relationship between age and the volumetric measurements and the difference between age groups and sexes was examined with the Mann Whitney U test. Males were coded as 1, and females as 0. The optimal cut-off values of the TV and RV for sex estimation were established from the reference dataset using the Youden’s index. Measurements equal to or above the determined cut-off value were classified as male (coded as 1); measurements below the cut-off value were classified as female (coded as 0). Receiver operating characteristic (ROC) curve analysis was performed. The discriminative ability for sex estimation of the determined cut-off values as well as the previously established cut-off values of the canine volume, 619 mm^3^ by De Angelis et al. [12], and 510 mm^3^ and 460 mm^3^ (for right and left side, respectively) by Tardivo et al., [1] were evaluated by area under the ROC curve (AUC), accuracy (Ac, i.e., overall percentage of correct predictions), sensitivity (Se, i.e., true positive rate considered to indicate the probability of the method correctly classifying males), specificity (Sp, i.e., true positive rate considered to indicate the probability of the method correctly classifying males), positive and negative predictive values (PPV and NPV), positive and negative likelihood ratios (LR + and LR-).

## Results

The ICC values for intra-observer agreement were 0.989 (95% CI; 0.974–0.995) and 0.984 (95% CI; 0.963–0.993) for TV and RV, respectively. It represents excellent agreement when greater than 0.90 [16]. Similarly, the ICC values for inter-observer agreement were 0.992 (95% CI; 0.982–0.997) for TV and 0.978 (95% CI; 0.948–0.990) for RV.

Out of 250 CBCT images, 168 (67.2%) were included in reference set and 82 (32.8%) were in validation set. Females and males were equally distributed in the reference set. The mean age of the sample ranged from 18 to 63 years old was 36.3 ± 11.7 in females and 36.9 ± 12.0 in males. In the reference sample, the mean ages of males and females were respectively 36.8 ± 11.9 and 37.0 ± 12.4. In the validation sample, they were 35.4 ± 11.5 and 36.7 ± 11.4. No statistically significant correlation between age and the maxillary canine’s TV and RV measurements nor difference was found between the age groups (*p* > 0.05).

The descriptive statistics of the TV and RV in males and females were presented in Table 2. It was found that the mean tooth and root volumes of the maxillary canine were significantly greater in males than in females (*p* < 0.05).

**Table 2:** Volumetric measurements of the maxillary canine in females and males

	Females		Males		
Measurements	Min – Max (mm^3^)	Mean ± SD (mm^3^)	Min – Max (mm^3^)	Mean ± SD (mm^3^)	*p*
*TV*	358–702	504 ± 78	447–1015	660 ± 116	0,000*
*RV*	180–408	278 ± 53	235–645	391 ± 77	0,000*

*TV*, tooth volume; *RV*, root volume

* *P* < 0.05

**Fig. 2:**
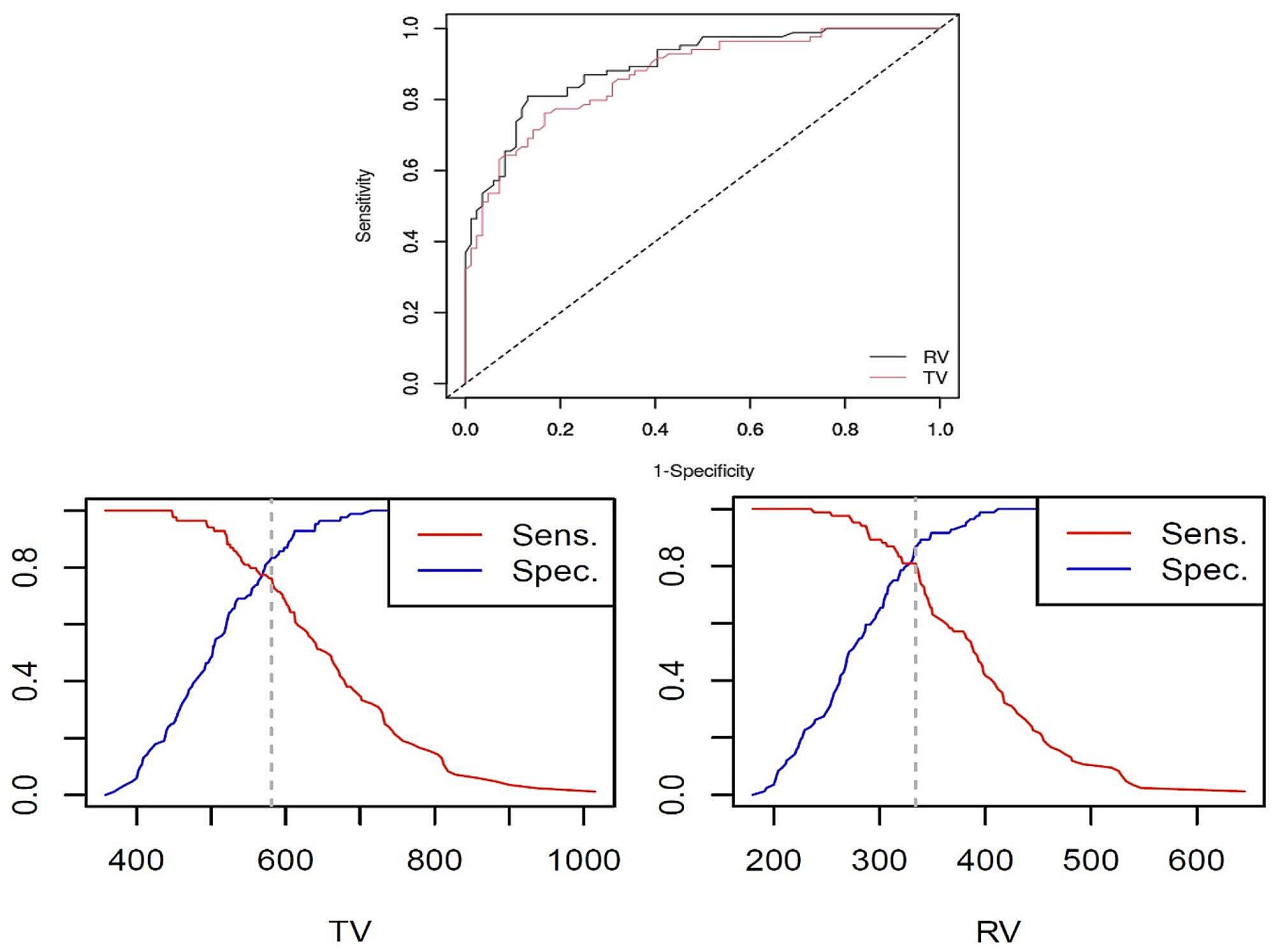
The ROC, sensitivity, and specificity curves of the optimal cut-off points of tooth volume (TV) and root volume (RV) for discriminating between females and males

The optimal cut-off values of 581 mm^3^ for TV and 334 mm^3^ for RV to discriminate the females and males were established. Their AUC values were 0.870 (95% CI, 0.818 to 0.922) and 0.896 (95% CI, 0.849 to 0.942), respectively (Fig. 2). Tables 3 and 4 presented respectively the contingency data and the performance measures of the derived cut-off values (TV ≥ 581 mm^3^ and RV ≥ 334 mm^3^) as well as those of the previous cut-off value (TV ≥ 619 mm^3^). For the other previously derived cut-off values of right TV ≥ 510 mm^3^ and left TV ≥ 460 mm^3^; AUC, Ac, Se, and Sp were found to be, respectively, 0.621 (95% CI, 0.551–0.690), 0.561 (95% CI, 0.447–0.670), 0.971 (95% CI, 0.847–0.999), and 0.271 (95% CI, 0.153–0.418).

**Table 3:** The contingency data showing discriminating performance of TV ≥ 581 mm^3^, RV ≥ 334 mm^3^ and TV ≥ 619 mm^3^ in both datasets

Sex	Reference set					Validation set									
	TV		Total	RV		Total	TV		Total	RV		Total	TV		Total
	≥ 581	< 581		≥ 334	< 334		≥ 581	< 581		≥ 334	< 334		≥ 619	< 619	
*Male*	64 ^TP^	20 ^FN^	84	68 ^TP^	16 ^FN^	84	26^TP^	8^FN^	34	28^TP^	6^FN^	34	24^TP^	10^FN^	34
*Female*	14 ^FP^	70 ^TN^	84	11 ^FP^	73 ^TN^	84	11^FP^	37^TN^	48	11^FP^	37^TN^	48	5^FP^	43^TN^	48
*Total*	78	90	168	79	89	168	37	45	82	39	43	82	29	53	82

*TV*, tooth volume; *RV*, root volume; *TP*, True positives; *FN*, False negatives; *FP*, False positives; *TN*, True negatives

**Table 4:** The discrimination performance measures of TV ≥ 581 mm^3^, RV ≥ 334 mm^3^ and TV ≥ 619 mm^3^ in both datasets

Measures	Reference set		Validation set		
	TV ≥ 581	RV ≥ 334	TV ≥ 581	RV ≥ 334	TV ≥ 619
*Ac*	0.798 (0.729–0.856)	0.839 (0.775–0.891)	0.768 (0.662–0.854)	0.793 (0.689–0.874)	0.817 (0.716–0.894)
*Se*	0.762 (0.657–0.848)	0.810 (0.709–0.887)	0.765 (0.588–0.893)	0.824 (0.655–0.932)	0.706 (0.525–0.849)
*Sp*	0.833 (0.736–0.906)	0.869 (0.778–0.933)	0.771 (0.627–0.880)	0.771 (0.627–0.880)	0.896 (0.773–0.965)
*PPV*	0.821 (0.718–0.889)	0.861 (0.765–0.919)	0.703 (0.541–0.858)	0.718 (0.560–0.883)	0.828 (0.656–0.918)
*NPV*	0.778 (0.676–0.871)	0.820 (0.724–0.905)	0.822 (0.670–0.910)	0.860 (0.715–0.931)	0.811 (0.665–0.933)
*LR+*	4.57 (2.79–7.48)	6.18 (3.53–10.83)	3.34 (1.92–5.79)	3.59 (2.09–6.18)	6.78 (2.88–15.97)
*LR-*	0.29 (0.19–0.42)	0.22 (0.14–0.34)	0.31 (0.16–0.57)	0.23 (0.11–0.48)	0.33 (0.19–0.56)

*TV*, tooth volume; *RV*, root volume; *Se*, sensitivity; *Sp*, specificity *PPV*, positive predictive value; *NPV*, negative predictive value; *LR+*, positive likelihood ratio; *LR-*, negative likelihood ratio; values are reported with 95% confidence intervals in the parenthesis.

## Discussion

Sexual dimorphism in tooth size, a well-known phenomenon, is explained by genetic point of view rather than role of sex hormones in dental development [17–20]. Differences in tooth size and in sexual dimorphism among prehistoric or modern populations have been widely reported in the literature [13, 14, 21–25]. This study confirmed a significant difference in the tooth and root volumes of maxillary canines between the sexes, with males exhibiting larger teeth, in contemporary Turkish individuals from Middle Anatolia. While various studies have explored tooth size as an indicator for sex estimation, recent advancements in imaging techniques, particularly CBCT, have offered new avenues for accurate measurements. The utilization of CBCT offers several advantages in developing sex estimation methods, primarily due to its increased usage in dentistry. This technology allows volumetric measurements of tooth tissues, including root volume, without the necessity for invasive procedures such as tooth extraction, offering a more accurate representation of size compared to linear measurements [7, 13]. The canines are reported to show the highest sexual dimorphism in most studies using linear measurements [3, 6, 24, 26–28]. Among the previous studies using volumetric measurement, some studies [7, 10], have found that mandibular canines show the most sex differences, while other has reported it’s the maxillary canines [1]. We conducted a pilot study using CBCT and assessed sex differences in dental tissue volumes and ratios (tooth volume, tooth volume without enamel, crown and root volumes, enamel, dentin and pulp volumes, crown/root, enamel/tooth, dentin/tooth, pulp/tooth volume ratios) of the maxillary and mandibular canines and second molars. Tooth volume, tooth volume without enamel, crown volume, and root volume exhibited statistically significant differences between the sexes for all teeth, while none of the ratios displayed a significant difference. Among these variables the tooth and root volumes of the maxillary canines emerged as the most distinctive and reliable indicators for sex estimation [29]. Therefore, the present study aimed to establish the cut-off values for the tooth and root volumes of the maxillary canines (TV ≥ 581 mm^3^ and RV ≥ 334 mm^3^) to improve accuracy of sex estimation and subsequently evaluate their performance in comparison with the previously proposed cut-off values of TV ≥ 619 mm^3^ by De Angelis et al. [12], and TV ≥ 510 mm^3^ (for right side) and TV ≥ 460 mm^3^ (for left side) by Tardivo et al. [1].

A prediction using diagnostic cut-off values inherently involves two types of errors: Type I errors (false positives, i.e., the definition of a female as a male) and Type II errors (false negatives, i.e., the definition of a male as a female). An ideal method minimizes both errors, requiring the determination of optimal cut-off values. The Se ( the probability of correctly classifying males) and Sp ( the probability of correctly classifying females) values are crucial indicators of diagnostic accuracy. In the current study, the derived cut-off value of TV ≥ 581 mm^3^ achieved predicting sex with 77% of the Se, Sp, and Ac values, while the previously established cut-off value of TV ≥ 619 mm^3^ were with respectively 0.71, 0.90 and 0.82. The new cut-off value of TV did not significantly improve the discrimination performance, exhibited a higher Se but considerably lower Sp and slightly lower Ac values. However, Sp of the cut-off value of TV ≥ 619 mm^3^ was considerably higher than Se, means that the value of 619 mm^3^ can successfully differentiate females in our population but, it may produce a significant false negative result. On the contrary, De Angelis et al. [12], in the original study conducted on 87 Italians aged 15 to 83 years, have reported higher Se value than Sp, indicated the value of 619 mm^3^ give a more accurate sex estimation for males. On the other hand, the correct prediction rate was similar for both studies. Regarding other previous cut-off values of right TV ≥ 510 mm^3^ and left TV ≥ 460 mm^3^, the correct prediction rate for our population (62%) did not achieve as high as those of the original study (85 − 84%) conducted on French individuals by Tardivo et al. [1]. Comparison with other study highlighted variations in statistical method and sex marker, Manhaes-Caldas et al. [7] have reported slightly lower performance with a cut-off value of 0.5 calculated with a specific model including the crown volume of the maxillary canine. The crown volume has showed 72% Se, 77% Sp, and 74% overall accuracy for sex estimation in Brazilian population. The differences among the results could be attributable to differences in populations as well as study methodology, sample size, and observer variations. Different magnitude of sex dimorphism is suggested among populations because of interactions of genetic and environmental influences [26, 27, 30]. The environmental factors, such as poor nutrition and recurrent illnesses, may play role in the variations in tooth size [21]. In addition, secular increase in tooth sizes have been reported in various populations, attributed to factors such as improvements in living conditions and nutritional status [31, 32]. Such variations due to ethnic, cultural, and socioeconomic factors may result in potential errors in the sex estimation. Accordingly, many researchers have emphasized the need for the development of population-specific standards for the sex estimation [5, 24, 25, 30]. The previous studies conducted on contemporary Turkish people from Turkey have focused on linear measurements (mesiodistal and/or buccolingual dimensions) of all teeth for sex estimation [14, 24]. İşcan and Kedici [24] created the discriminant functions for sex estimation using different combinations of teeth or variables and observed that the maxillary canine, the mandibular canine and second molar contributed the most to these models. The accuracy achieved in between 73% and 77%. Ateş et al. [14] determined that dimensions of the mandibular canine, maxillary canine and second incisor served as discriminating variables, when using anterior teeth for sex estimation. The classification accuracy of the discriminant functions was found to be 76% for the maxillary anterior teeth and 81% for the mandibular anterior teeth. These accuracy rates are similar to our result, however, there are differences in the used measures (linear measurements) and statistical models between the studies. Hence, the applicability of the volumetric method across various populations or within the populations can be examined in further studies.

The present study included intact teeth of individuals aged from 18 to 63 years to avoid any potential source of bias for sex estimation. Regarding physiological attrition, severe worn teeth, were not included to the study sample. Furthermore, no significant relationship between age and the examined parameters was found. This indicates that this method holds consistent applicability across different age groups. However, in advanced age, the probability of encountering severe attrition increases, impacting the tooth size and altering the TV measurements, which may diminish the method’s applicability of for older individuals. On the other hand, cement deposition, another age-related change, could potentially compensate for the volume reduction caused by attrition [33]. However, this possibility requires examination, and the cut-off value of TV can be tested in future studies, especially for severe worn teeth. As an alternative to TV, for the worn or decayed tooth, the cut-off value of the RV can be used to estimate sex. Moreover, the cut-off value of RV ≥ 334 mm^3^ demonstrated superior performance compared to TV ≥ 581 mm^3^, but still could not exceed 80% Ac and provided slightly more accurate predictions for males with a higher Se value (82%) than Sp (77%). This is lower than a previous study [13] where, like our study, root volume for sex determination was assessed using CT, and an accuracy of 97% was achieved with the discriminant function including maxillary canine. This discrepancy may be attributed to the difference in the populations, which Kazzazi et al.’s [13] study sample were consisted of Iranian archaeological subjects.

This study attempts to establish forensic standards for sex estimation in Turkish individuals using a simple method that involves a single tooth and volumetric measurement of the maxillary canine on CBCT images. The performance of dental volumetric techniques for sex estimation was not improved with the derived cut-off values of volumes of the maxillary canines and remained still lower than those achieved with other osteometric techniques. The method partially meets the Mohan and Daubert criteria [34], indicating that the techniques used in forensic investigations should provide a minimum of 80% accuracy and a maximum of 10% intra-observer error rate for admissibility in a court of law, as it presents excellent intra-observer reliability but borderline accuracy value.

Although the volumetric measurements can be more time-consuming and challenging than linear measurements, De Angelis et al. [12] reported that the application of the method is very simple to be achieved by everyone and that more tools to measure tooth volume is also available, such as a test tube filled by water, which can also apply for root volume. The study is not without limitations. The sample size, although comprehensive, may not fully represent the entire population’s diversity. The factors of tooth position or impaction, which could potentially influence TV measurements and, in turn, the performance of the method, were not considered. Additionally, the study’s findings might be influenced by the used software and measurement techniques.

## Conclusions

The method based on the total tooth and root volumes in the maxillary canines can be useful in case where maxillary canine is single evidence and/or its crown is destructed, to estimate sex with moderate accuracy [35] in the field of forensic sciences and anthropology. The cut-off values of TV and RV in this method were respectively 581 mm^3^ and 334 mm^3^ for contemporary Turkish individuals. Future research could explore the method’s applicability to different populations and assess the method’s precision and robustness across various measurement techniques.

## Data Availability

The datasets generated and/or analysed during the current study are available from the corresponding author upon reasonable request.

## Abbreviations

Ac: Accuracy
AUC: Area Under the ROC Curve
CBCT: Cone Beam Computed Tomography
FN: False Negative
FP: False Positive
LR: Likelihood Ratio
NPV: Negative Predictive Values
PPV: Positive Predictive Values
ROC: Receiver Operating Characteristic
ROI: Region of interest
RV: Root Volume
Se: Sensitivity
Sp: Specificity
TN: True Negative
TP: True Positive
TV: Tooth Volume
